# Comparative study of laparoscopic ventral mesh rectopexy versus perineal stapler resection for external full-thickness rectal prolapse in elderly patients: enhanced outcomes and reduced recurrence rates—a retrospective cohort study

**DOI:** 10.1007/s10151-024-02919-1

**Published:** 2024-04-15

**Authors:** T. A. A. M. Habeeb, M. Podda, M. Chiaretti, A. Kechagias, J. B. Lledó, Abd-Elfattah Kalmoush, Fawzy M. Mustafa, Mohammed Shaaban Nassar, Mohamed fathy Labib, Sobhy rezk ahmed Teama, Mohammed Hassan Elshafey, Hamdi Elbelkasi, Mohamed Ibrahim Abo Alsaad, Ahmed M. Sallam, Hassan Ashour, Mohamed Ibrahim Mansour, Abdelshafy Mostafa, Tamer Mohamed Elshahidy, Ahmed m. Yehia, Tamer Rushdy, Alaaedin Ramadan, Abd Elwahab M. Hamed, Mahmoud Abdou Yassin, Abd-Elrahman M. Metwalli

**Affiliations:** 1 Department of General Surgery, Faculty of Medicine, Zagazig University, 1 Faculty of Medicine Street, Zagazig, Sharqia Egypt; 2 Department of Surgical Science, Cagliari University Hospital, Monserrato, 09042 Cagliari, Italy; 3 Paride Stefanini General and Specialist Surgery Department, Sapienza University of Rome IT, Rome, Italy; 4 Department of Gastroenterology and Alimentary Tract Surgery, Tampere, Finland; 5 Department of Surgery, La Fe University Hospital, Valencia, Spain; 6 General Surgery Department, Faculty of Medicine, Al-Azher University, Cairo, Egypt; 7 General Surgery Department, Mataryia Teaching Hospital (GOTHI), Cairo, Egypt; 8 General Surgery Department, Faculty of Medicine, Merit University, Sohag, Egypt

**Keywords:** Elderly, Functional outcomes, Laparoscopic ventral mesh rectopexy, Perineal stapler resection, Recurrence, Rectal prolapse

## Abstract

**Background:**

In elderly patients with external full-thickness rectal prolapse (EFTRP), the exact differences in postoperative recurrence and functional outcomes between laparoscopic ventral mesh rectopexy (LVMR) and perineal stapler resection (PSR) have not yet been investigated.

**Methods:**

We conducted a retrospective multicenter study on 330 elderly patients divided into LVMR group (*n* = 250) and PSR (*n* = 80) from April 2012 to April 2019. Patients were evaluated before and after surgery by Wexner incontinence scale, Altomare constipation scale, and patient satisfaction questionnaire. The primary outcomes were incidence and risk factors for EFTRP recurrence. Secondary outcomes were postoperative incontinence, constipation, and patient satisfaction.

**Results:**

LVMR was associated with fewer postoperative complications (*p* < 0.001), lower prolapse recurrence (*p* < 0.001), lower Wexner incontinence score (*p* = 0.03), and lower Altomare’s score (*p* = 0.047). Furthermore, LVMR demonstrated a significantly higher surgery–recurrence interval (*p* < 0.001), incontinence improvement (*p* = 0.019), and patient satisfaction (*p* < 0.001) than PSR. Three and 13 patients developed new symptoms in LVMR and PSR, respectively. The predictors for prolapse recurrence were LVMR (associated with 93% risk reduction of recurrence, OR 0.067, 95% CI 0.03–0.347, *p* = 0.001), symptom duration (prolonged duration was associated with an increased risk of recurrence, OR 1.131, 95% CI 1.036–1.236, *p* = 0.006), and length of prolapse (increased length was associated with a high recurrence risk (OR = 1.407, 95% CI = 1.197–1.655, *p* < 0.001).

**Conclusions:**

LVMR is safe for EFTRP treatment in elderly patients with low recurrence, and improved postoperative functional outcomes.

**Trial registration:**

Clinical Trial.gov (NCT05915936), retrospectively registered on June 14, 2023.

## Introduction

The external full-thickness rectal prolapse (EFTRP) or procidentia is the complete protrusion of the rectal wall through the anus. Because of social embarrassment, it is underreported and underestimated [1]. EFTRP leads to considerable patient distress, social isolation, low self-esteem, embarrassment, and can result in life-threatening complications due to strangulation [2]. Chronic constipation affects up to 70% of patients with EFTRP, while fecal incontinence ranges from 50% to 88% [3]. In elderly patients with EFTRP, the surgical risks must be carefully managed because they are frequently associated with additional comorbidities [1]. Many authors have described both perineal and abdominal techniques. However, an optimal surgical approach has yet to be established [1, 4]. Typically, perineal procedures are performed in patients with limited surgical tolerance under spinal anesthesia but are associated with higher recurrence and postoperative fecal incontinence (FI) rates [5]. Delorme and Altemeier are two commonly performed perineal techniques. In 2008, Scherer et al. introduced perineal stapled prolapse resection (PSP) using a Contour Transtar stapler. Romano et al. modified this technique, and it has gained popularity [6, 7]. Patients with adequate metabolic, cardiological, and respiratory reserve undergo the laparoscopic procedure despite longer operative times, more invasive approach, and carrying a higher risk of surgical complication rate than perineal operations. Laparoscopic approaches offer cost reduction owing to shorter hospital stays, faster patient recovery, and decreased morbidity [8]. Laparoscopic ventral mesh rectopexy (LVMR) is an autonomic nerve-sparing technique that has gained popularity owing to its favorable postoperative functional outcomes, low recurrence rate, and low morbidity [9, 10]. Advancements in laparoscopy and general anesthesia have expanded the application of abdominal rectopexy to include elderly patients [10]. This study aimed to evaluate the incidence and risk factors of recurrent prolapse (RP), postoperative fecal incontinence (FI), postoperative obstructive defecation syndrome (ODS), and patient satisfaction between LVMR and PSR in elderly patients with EFTRP.

## Materials and methods

### Study design and eligibility criteria

Patients included in the cohort were treated according to a previously established protocol created under the Helsinki Declaration, approved by the Zagazig Ethics Committee [10886], and registered at Clinical Trial.gov (NCT05915936). The results were reported according to the Guidelines for Strengthening the Reporting of Observational Studies in Epidemiology (STROBE) [11]. All patients provided consent prior to surgery. From April 2012 to April 2019, 330 elderly patients with complete external EFTRP (Oxford prolapse grade 5) [12] were surgically treated with LVMR or PSR. A cumulative database collected by the personnel of colorectal surgery units at university hospitals (University of Cagliari, Hospital Universitari i Politècnic la Fe, Valencia, Spain, and four hospitals in Egypt) was retrospectively analyzed. The inclusion criteria comprised patients with EFTRP who completed at least 4 years of follow-up, aged ≥ 60 years [13], both sexes, and American Society of Anesthesiologists (ASA) score I–III. The exclusion criteria were age < 60 years, incomplete medical records, concurrent colorectal procedures, multicompartmental prolapse requiring combined operations, open abdominal rectopexy, megacolon, pregnancy, inflammatory bowel disease, unfit for general anesthesia, recurrent rectal prolapse, prior anal or pelvic surgery, systemic steroid therapy, connective tissue disease, abnormal thyroid function, diverticulosis/stricture of the colon, previous colorectal resection surgery, neurological disease, psychiatric disorders, and chronic opioid use.

### Outcome definitions and measurements

The primary outcomes were the incidence and risk factors for recurrent EFTRP after LVMR and PSR. The secondary outcomes included postoperative FI, ODS, and patient satisfaction. Postoperative recurrence was detected by physical examination, anorectal manometry, defecography, or dynamic pelvic magnetic resonance imaging (MRI), which were performed selectively according to availability in our hospitals. Recurrent procidentia is a very severe form of prolapse seen even with a simple clinical examination; in the case of recurrent procidentia, anal manometry, defecography, and MRI were used in some patients as an adjunctive tool to objectively quantify the weakness of the sphincter and when further differentiation in diagnosis may be needed to identify dyssynergia of the pelvic floor muscles. Follow-up examinations were performed by one surgeon who recorded recurrence of EFTRP. Complete rectal prolapse showed concentric folds of the rectal muscle. The length of the rectal prolapse was measured with the patient sitting on a commode. Preoperative and postoperative continence status was assessed using the Jorge–Wexner grading scale [14], encompassing five items with a total score ranging from 0 (complete continence) to 20 (complete incontinence). To assess ODS, preoperative and postoperative Altomare scores were assessed using an eight-point Likert scale [15]. Postoperative morbidity was assessed using the Clavien–Dindo classification [16]. EFTRP was measured by assessing the distance between the distal margin of the rectum and the anal margin during stool straining. Anal stenosis is defined as narrowing of the anal orifice and rectal canal that cannot be explored with either a 12-mm colonoscopy or an exploratory finger [17]. Patient satisfaction was rated as satisfied or dissatisfied. Dolichocolon is the presence of a redundant colon (verified by preoperative colonoscopy and confirmed during surgery) [18]

### Perioperative technique

**Fig. 1:**
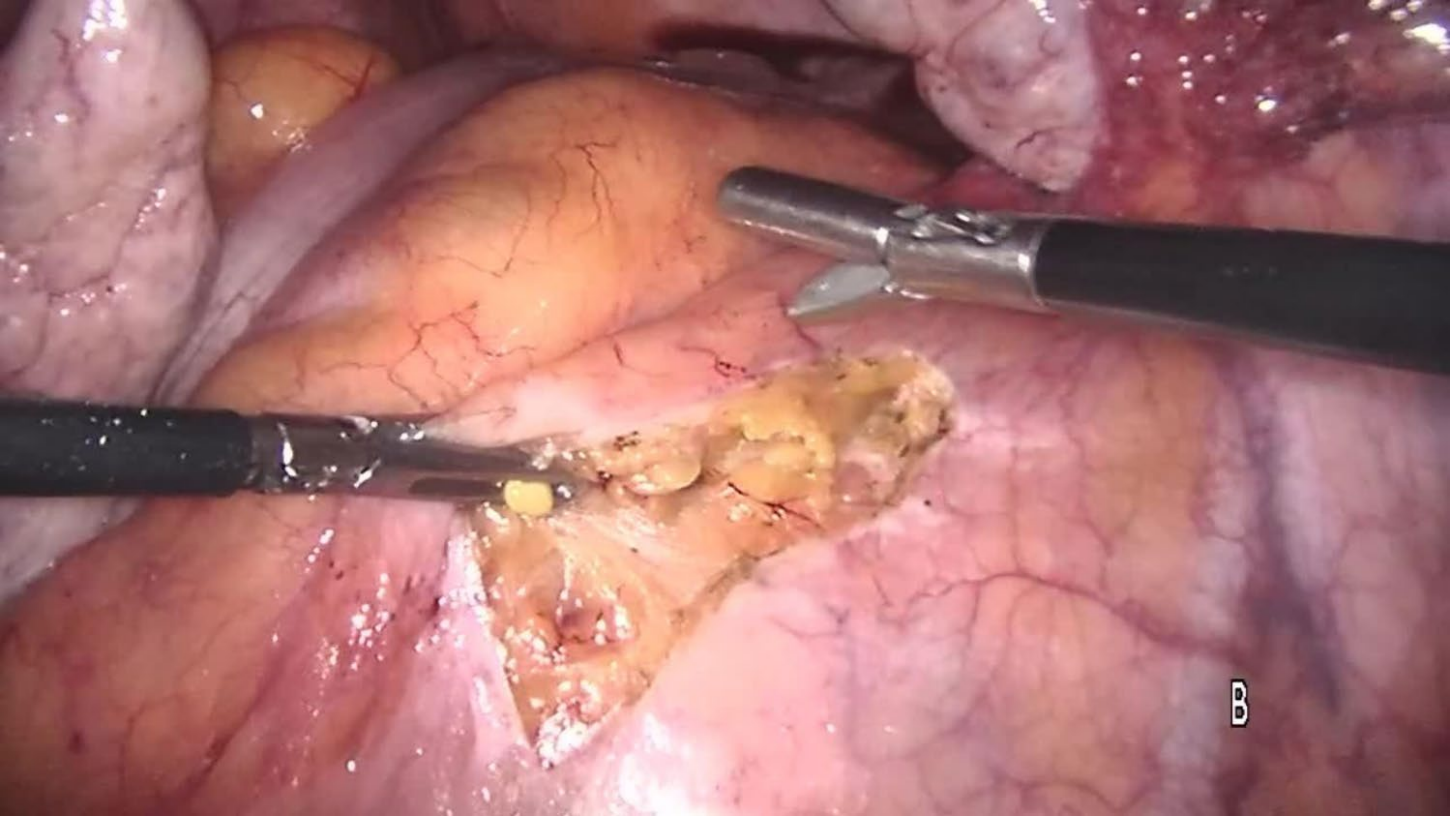
Incision of the peritoneum starts at the sacral promontory and extends downwards in L-shape

**Fig. 2:**
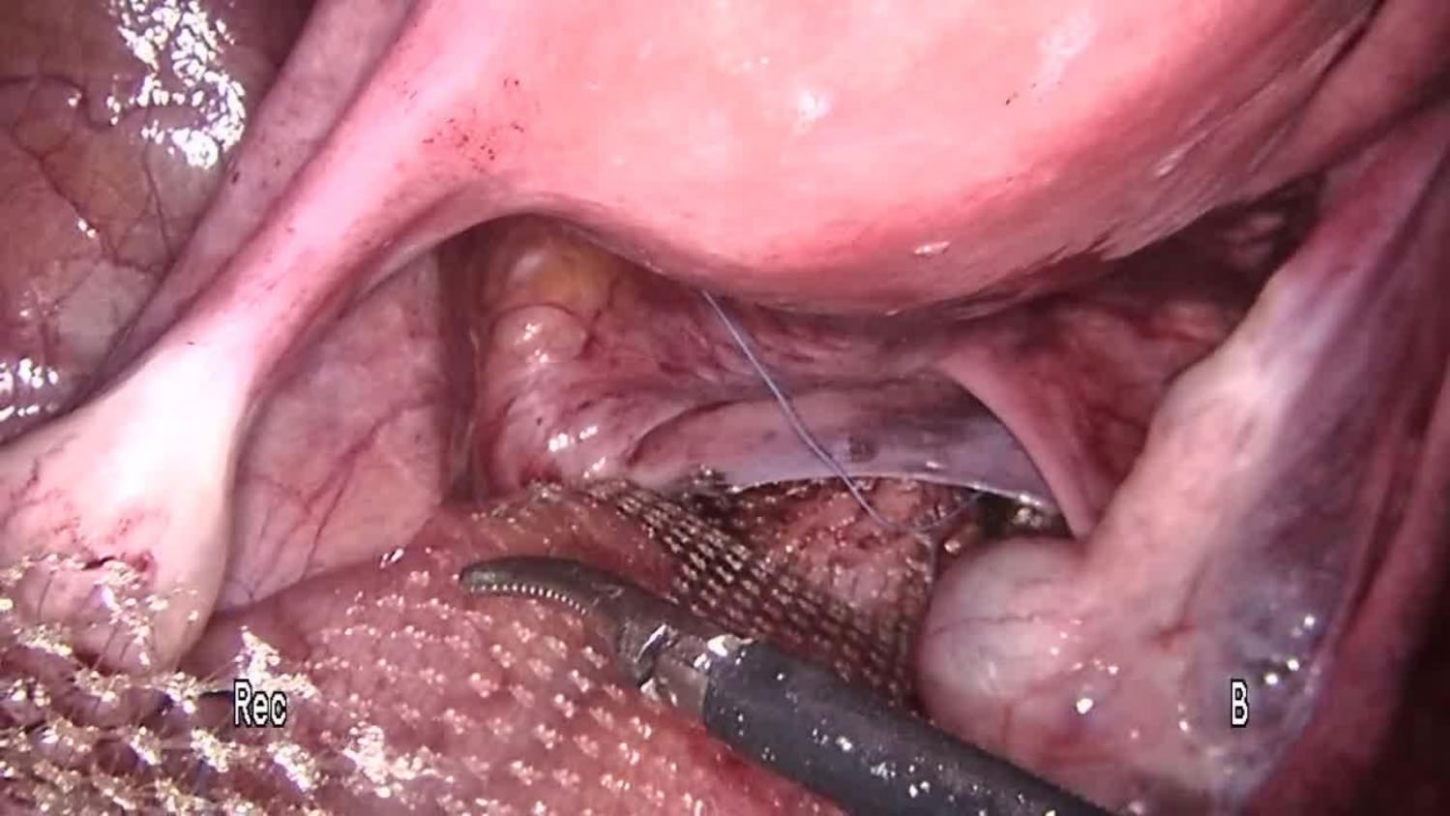
The polypropylene mesh is fixed to the anterior wall of the rectum and to the pelvic floor muscle

**Fig. 3:**
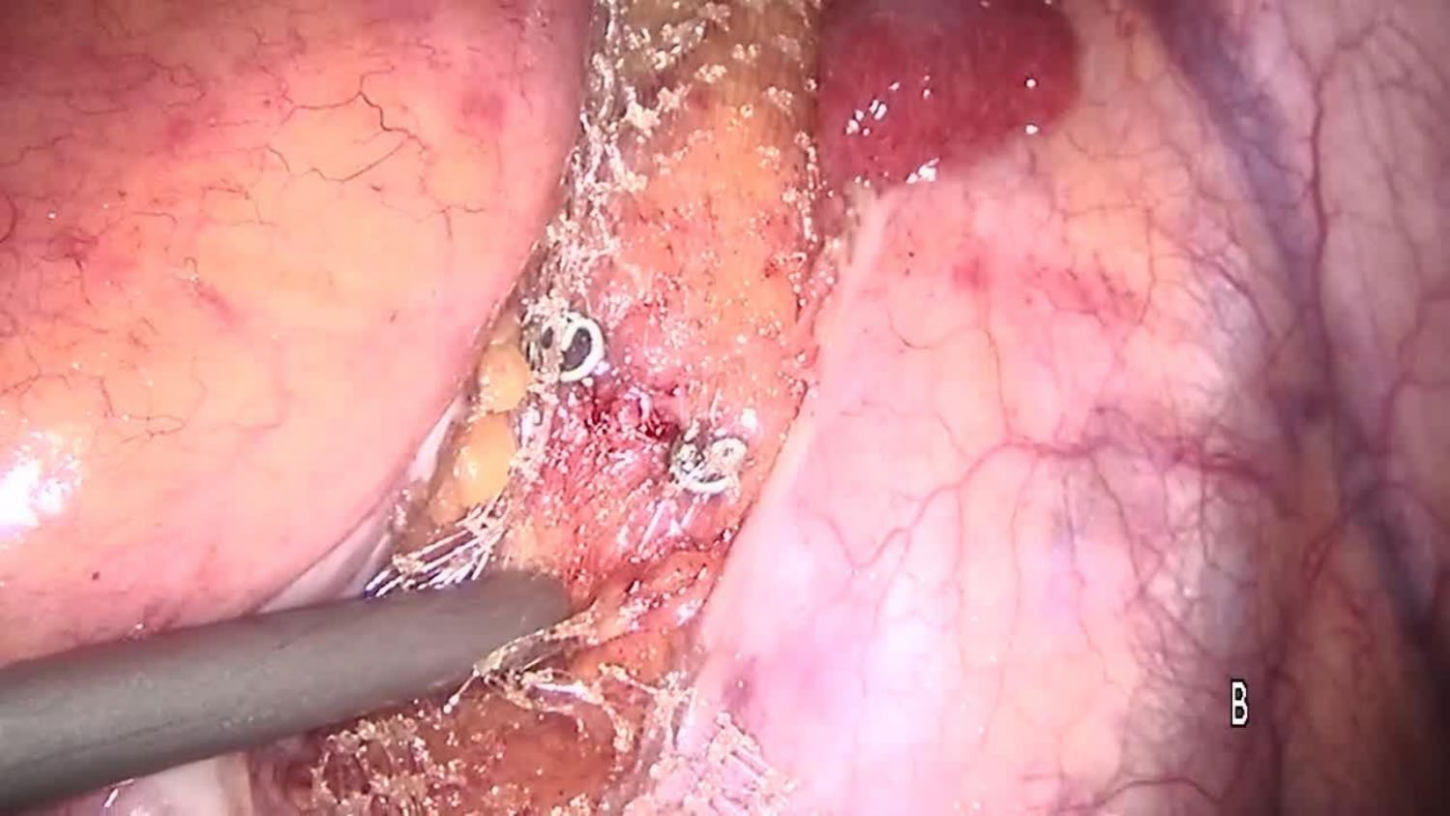
The mesh is fixed to the sacral promontory by a titanium helical tacker

**Fig. 4:**
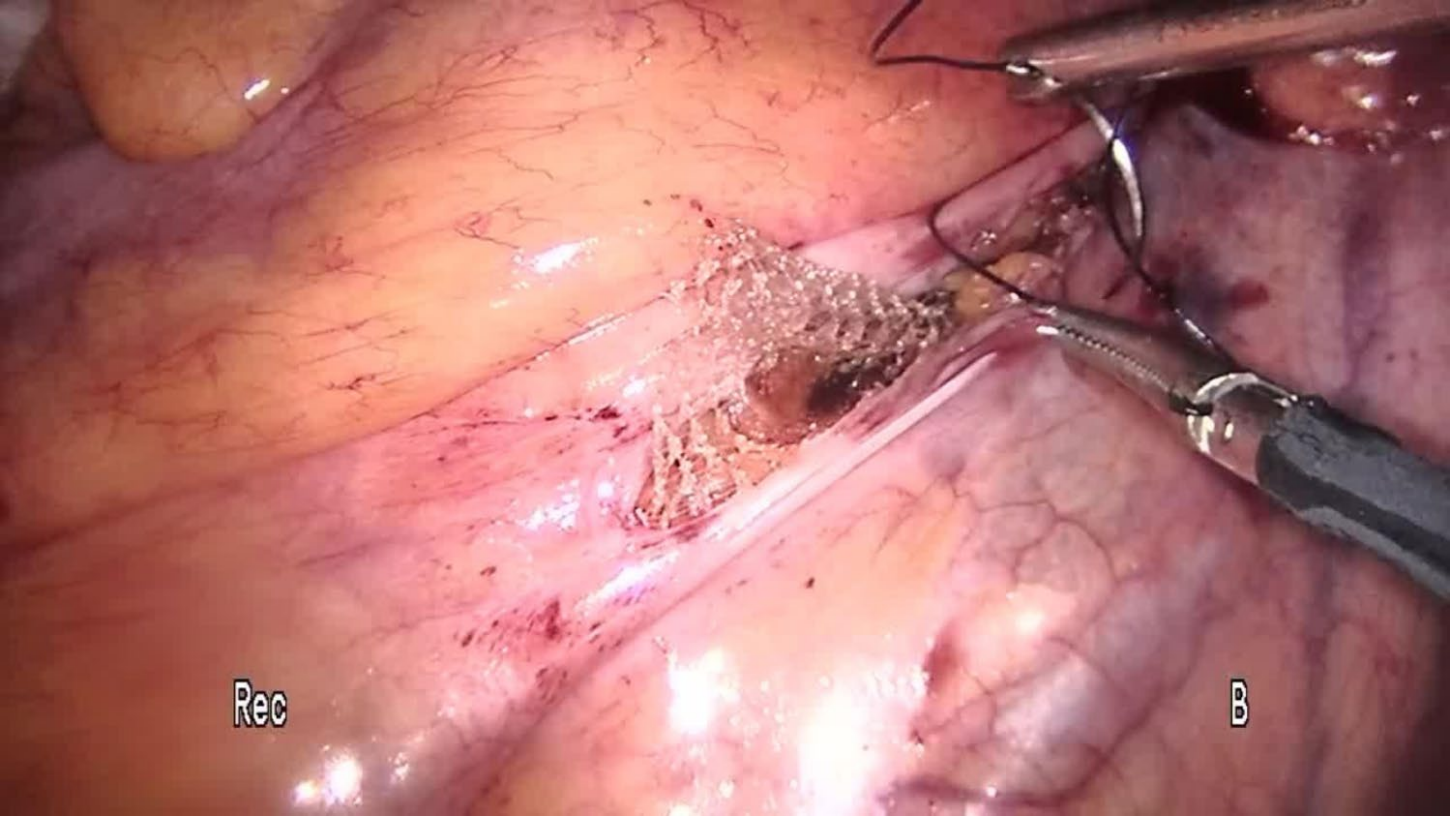
We cover the mesh with the peritoneum to avoid mesh exposure and subsequent complications

Perioperative management was the same in all the centers. The preoperative workup consisted of a thorough clinical history, physical examination (straining maneuver), colonoscopy (to exclude intraluminal pathology), magnetic resonance defecography (to evaluate sphincter integrity and rule out cystocele or enterocoele), or anal manometry (to evaluate the functioning of the anorectal complex) according to the guidelines [3, 19–22] and were performed selectively according to availability in our hospitals. All procedures and follow-up were performed with the same technique in each team, ensuring standardization of procedures, uniformity of data collection and follow-up procedures, comprehensive data validation, and quality control process. The choice of surgical treatment was tailored according to the surgeon’s preference and experience. Bladder evacuation was routinely performed by catheterization. The surgical technique for PSR has been previously described [23]. Under spinal anesthesia, a slight Trendelenburg position was chosen to free the pouch of Douglas from any deep enterocele. In female patients, the stapler was fired after the digital exploration of the back wall of the vagina to exclude entrapment. A careful bi-manual examination was performed, to exclude the entrapment of any intraperitoneal organ in the prolapse. The prolapse was longitudinally incised at the 3 and 9 o’clock positions using a green cartridge linear stapler (linear cutter 75–100 mm, Ethicon). The resection was completed by applying the stapler extended 1–2 cm and parallel to the dentate line. The stapled resection line was oversewn using absorbable polyglactin (Vicryl) sutures 2/0 to ensure hemostasis and reinforcement of the anastomosis. Laparoscopic ventral mesh rectopexy (LVMR) was performed as described before [24]. Under general anesthesia, three ports were utilized: the optic 10-mm one in the right iliac fossa, an operative 5-mm port in the right upper quadrant, and a 5-mm port in the suprapubic region. Dissection started at the sacral promontory (Fig. 1) and extended in an L-shape on the side of the rectum until the rectum. A purely anterior rectal dissection is then undertaken in this areolar tissue to create a 4- or 5-cm-wide pocket from the depth of the pouch of Douglas to the level of the pelvic floor muscle. A 3 × 20-cm strip of polypropylene mesh (polypropylene knitted nonabsorbable undyed, Ethicon, Johnson & Johnson) was fixed to pelvic floor muscle (levator ani) [25] and sutured on the anterior surface of the rectum using two parallel rows of interrupted nonabsorbable sutures (Ethibond Excel 00, Ethicon, Johnson & Johnson, Wokingham, Surrey, UK) (Fig. 2). The mesh was anchored to the right sacral promontory using tacker titanium (CapSure, Bard Inc, USA) (Fig. 3). Complete coverage of the mesh by the peritoneum was achieved using a 2/0 Vicryl absorbable continuous suture (Fig. 4). Patients were discharged within 2–5 days after surgery according to discharge criteria that encompassed the absence of suspected symptoms, feeding and ambulation recovery, and no need for additional treatment related to comorbidities. Clinical checks were scheduled at the first, third, and twelfth month after surgery, then yearly until the end of the follow-up period and consisted of either an outpatient clinic interview or a telephone interview considering the elderly age of patients.

### Statistical analysis

Data management and statistical analyses were performed using IBM SPSS Statistics, version 28 (IBM, Armonk, New York, USA). The normality of the quantitative data was assessed using the Kolmogorov–Smirnov test and direct data visualization techniques. On the basis of normality assessment, quantitative data were summarized using means with standard deviations or medians with ranges. Continuous data were presented as frequencies and percentages. Independent *t* tests or Mann–Whitney *U* tests were used for normally and non-normally distributed numerical variables to compare quantitative data between the groups under investigation. Categorical data were compared using the chi-square test or Fisher’s exact test. Recurrence-free survival was estimated using Kaplan–Meier analysis, and the log-rank test was used to compare the Kaplan–Meier curves. Univariate level and variables that are known to be clinically relevant were included in a stepwise multivariate logistic regression model. Odds ratios with 95% confidence intervals were calculated. All statistical tests were two-sided, and statistical significance was set at *p* < 0.05.

## Results

**Fig. 5:**
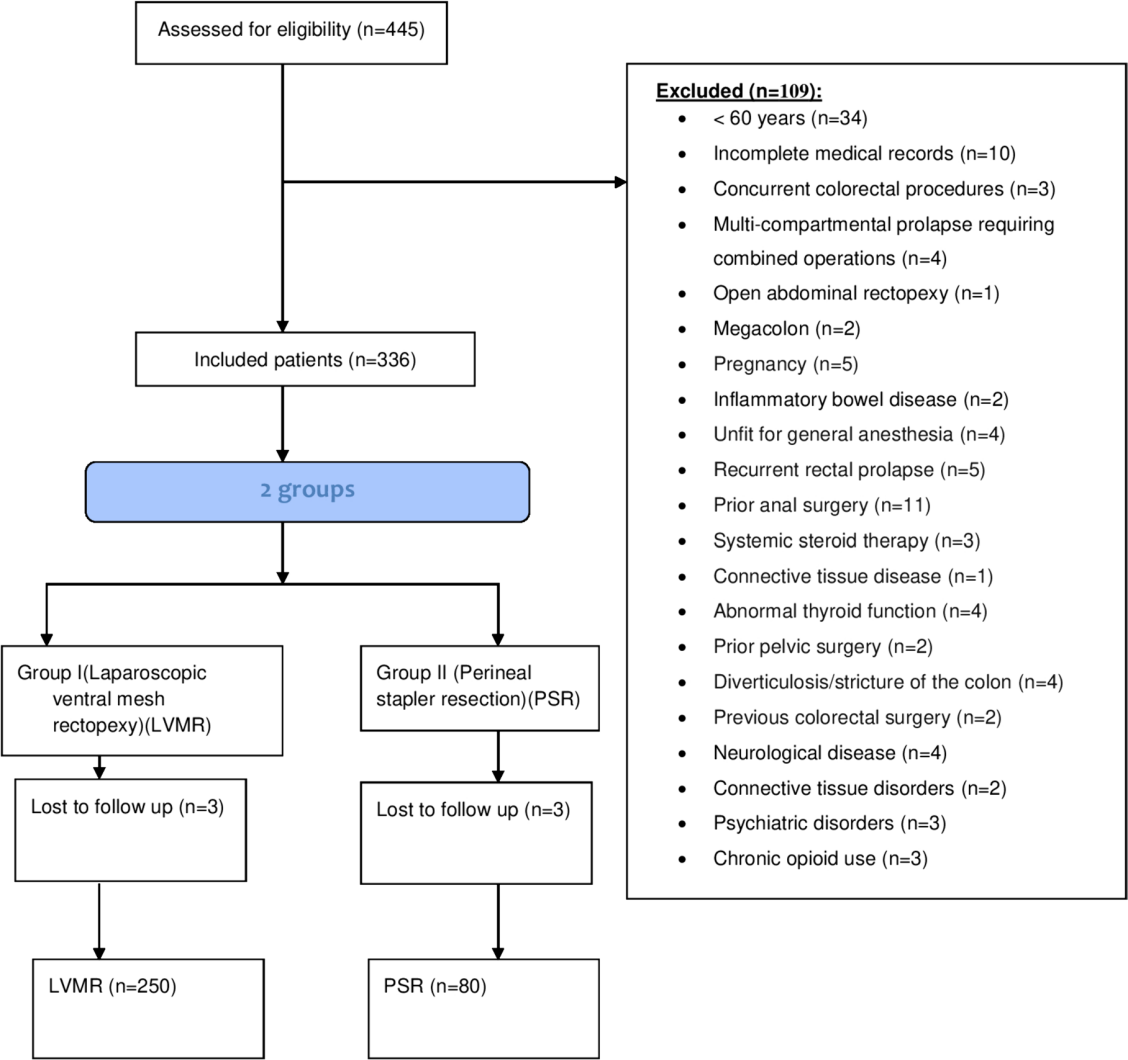
Flowchart of inclusion and exclusion criteria of the studied groups

Figure 5 shows the flowchart of the inclusion and exclusion criteria. The demographics and baseline characteristics of both groups [group I (LVMR *n* = 250), and group II (PSR *n* = 80)] are shown in Table 1. There were no significant differences in age (*p* = 0.532), sex (*p* = 0.585), parity (*p* = 0.883), body mass index (*p* = 0.994), initial symptoms (*p* = 0.930), prolapse length (*p* = 0.223), ASA (*p* = 0.454), diabetes mellitus (*p* = 0.116), coronary disease (*p* = 0.327), and median preoperative maximum squeeze pressure on manometry (*p* = 0.158). However, patients in group II (PSR) had significantly longer symptoms duration (*p* < 0.001), a higher incidence of dolichocolon (*p* < 0.001), and higher median resting pressure on preoperative manometry (*p* = 0.00).

**Table 1:** Demographics and baseline characteristics of the studied groups

	Group I (LVMR) (*n* = 250)	Group II (PSR) (*n* = 80)	*p *value
Age (years), mean ± SD	68 ± 6	68 ± 6	0.532
Sex,* n* (%)			0.585
Male	54 (21.6%)	15 (18.8%)	
Female	196 (78.4%)	65 (81.3%)	
Parity,* n* (%)			0.883
Nulliparous	14 (7.1%)	5 (7.7%)	
Multiparous	182 (92.9%)	60 (92.3%)	
Body mass index, mean ± SD	30 ± 6	30 ± 6	0.994
Initial symptoms*, n* (%)			0.930
Prolapse only	87 (34.8%)	26 (32.5%)	
Prolapse with incontinence	111 (44.4%)	37 (46.3%)	
Prolapse with OD	52 (20.8%)	17 (21.3%)	
Symptoms duration (months)			< 0.001*
Median (range)	11 (3–41)	18 (4–38)	
Length of prolapse (cm)			0.223
Median (range)	11 (5–31)	11 (5–32)	
ASA,* n* (%)			0.454
ASA I	34 (13.6%)	7 (8.8%)	
ASA II	194 (77.6%)	64 (80%)	
ASA III	22 (8.8%)	9 (11.3%)	
Diabetes mellitus, *n* (%)	144 (57.6%)	54 (67.5%)	0.116
Coronary diseases,* n* (%)	177 (70.8%)	52 (65%)	0.327
Dolichocolon,* n* (%)	4 (1.6%)	9 (11.3%)	< 0.001*
Preoperative manometry,* n* (%)	139 (55.6%)	40 (50%)	0.382
Resting pressure (median), mmHg	81 (26–90)	82 (78–87)	0.000*
Maximum squeeze pressure (median), mmHg	78 (34–100)	73 (47–92)	0.158

*OD* obstructed defecation

*Significant *p* value

Table 2 shows that there are no significant difference among groups I and II regarding intraoperative findings except LVMR’s longer operative time in comparison with PSR (*p* < 0.001). Only three patients in the LVMR group experienced bleeding, while two had bleeding, and one had mechanical staple dysfunction in the PSR group. No conversion was reported for LVMR. The mean number of stapler cartridges used in the PSR group was 5 ± 1.

**Table 2:** Intraoperative findings in the studied groups

	Group I (LVMR) (*n* = 250)	Group II (PSR) (*n* = 80)	*p* value
Operative time (min), mean ± SD	90 ± 9	36 ± 9	< 0.001*
Blood loss (ml), median (range)	99 (29–392)	100 (29–292)	0.162
Intraoperative complications,* n* (%)	3 (1.2)	3 (3.8)	0.156
Type of complications,* n* (%)**			
Bleeding	3 (100%)	2 (66.7%)	
Mechanical staple dysfunction	0 (0%)	1 (33.3%)	NA
Conversion,* n* (%)	0 (0)	–	–
Number of stapler cartridges used ± SD		5 ± 1	–

*NA* not applicable

*Significant *p* value

**Percentages were calculated on the basis of those with intraoperative complications

**Fig. 6:**
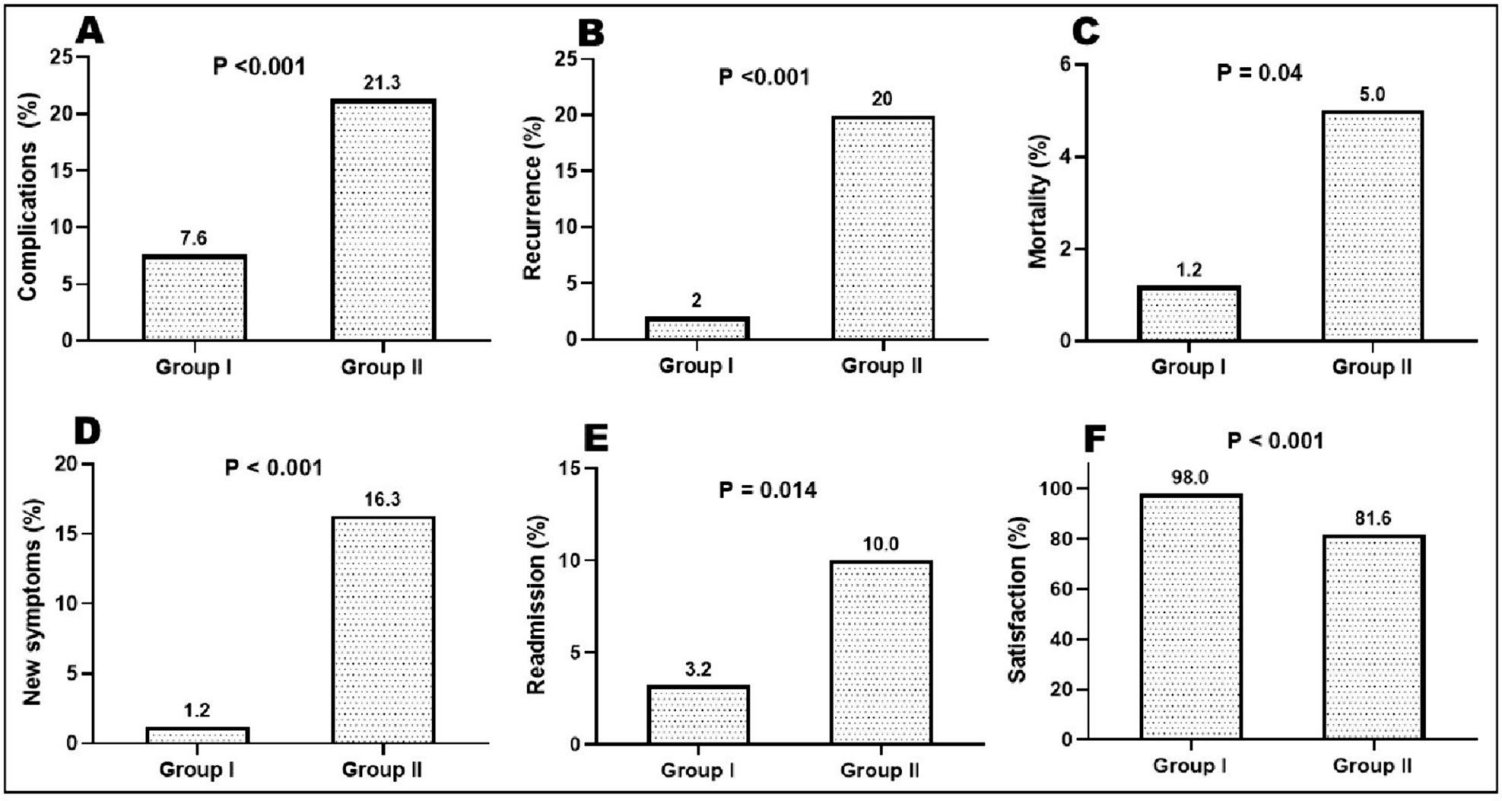
Main postoperative outcomes: **a** complications; **b** recurrence; **c** mortality; **d** new symptoms; **e** readmission; **f** satisfaction

Postoperative data demonstrated that LVMR showed significantly better outcomes in terms of length of hospital stay (*p* < 0.001), postoperative complications (*p* < 0.001), hospital readmission within 30 days (*p* = 0.014), and mortality (*p* = 0.04). Postoperative complications occurred in 19 patients (7.6%) with LVMR and 17 patients (21.3%) with PSR (*p* < 0.001). Complications with Clavien–Dindo score higher than grade III were experienced by three patients (15.8%) in the LVMR group and two (11.8%) in the PSR group (Table 3, Fig. 6).

**Table 3:** Postoperative data in the studied groups

	Group I (LVMR) (*n* = 250)	Group II (PSR) (*n* = 80)	*p* value
Hospital stay (days), median (range)	2 (2–5)	5 (2–12)	< 0.001*
Postoperative complications,* n* (%)	19 (7.6%)	17 (21.3%)	< 0.001*
Readmission within 30 days,* n* (%)	8 (3.2%)	8 (10%)	0.014*
Mortality,* n* (%)	3 (1.2%)	4 (5%)	0.040*
Type of postoperative complications,* n* (%)**	19 patients (7.6%)	17 patients (21.3%)	< 0.001*
Adhesive intestinal obstruction	1 (5.3%)	0 (0%)	NA
Deep venous thrombosis	1 (5.3%)	1 (5.9%)	
Infection at the staple line	0 (0%)	5 (29.4%)	
Intra-abdominal abscess	1 (5.3%)	0 (0%)	
Mesh-related complication	3 (15.8%)	0 (0%)	
Myocardial infarction	1 (5.3%)	0 (0%)	
Pelvic hematoma	1 (5.3%)	0 (0%)	
Port site hernia	2 (10.5%)	0 (0%)	
Prolonged ileus	1 (5.3%)	0 (0%)	
Pulmonary infection	1 (5.3%)	1 (5.9%)	
Rectal stenosis with fecal impaction	2 (10.5%)	0 (0%)	
Staple line dehiscence with abscess	0 (0%)	2 (11.8%)	
Stapler line bleeding	0 (0%)	3 (17.6%)	
Urinary fistula	1 (5.3%)	0 (0%)	
Urinary retention	0 (0%)	5 (29.4%)	
Urinary tract infection	1 (5.3%)	0 (0%)	
Wound infection	3 (15.8%)	0 (0%)	
Treatment of complications,* n* (%)**			
Conservative treatment	10 (52.6%)	13 (76.5%)	
Endoscopic dilatation	2 (10.5%)	0 (0%)	
Radiological intervention	1 (5.3%)	0 (0%)	
Surgical re-intervention	6 (31.6%)	4 (23.5%)	NA
Indication for re-intervention			
Port site hernia	2	0	
Mesh related complications	1	0	
Pelvic hematoma	1	0	
Urinary fistula	1	0	
Adhesive IO	1	0	
Stapler line bleeding	0	2	
Stapler line dehiscence with pararectal abscess	0	2	
Clavien–Dindo classification,* n* (%)**			
I	3 (15.8%)	4 (23.5%)	
II	4 (21.1%)	6 (35.3)	
III-A	5 (26.3%)	5 (29.4%)	
III-B	4 (21.1%)	0 (0%)	
IV	1 (5.3%)	1 (5.9%)	
V	2 (10.5%)	1 (5.9%)	NA

Percentages were calculated based on those with intraoperative complications

*NA* not applicable

*Significant *p* value

Table 4 shows the postoperative functional outcomes and patient satisfaction. LVMR demonstrated a significantly lower recurrence rate (RR) than PSR, with 2% and 20% RR, respectively (*p* < 0.001). The length of recurrence was also significantly shorter in the LVMR group (69 ± 9 mm) than in the PSR group (96 ± 14 mm) (*p* < 0.001). In terms of functional outcomes, LVMR showed superior results, as indicated by lower 3-month and 4-year Wexner incontinence scores (median 1 vs. 2, *p* = 0.023 and *p* = 0.03, respectively) and 3-month and 4-year Altomare scores (median 0 vs. 1, *p* = 0.025 and *p* = 0.047, respectively) compared to PSR. Furthermore, LVMR demonstrated a significantly longer surgery–recurrence interval (40 ± 10 months vs. 13 ± 9 months, *p* < 0.001), higher rates of incontinence improvement (95.5% vs. 83.8%, *p* = 0.019), higher patient satisfaction (98% vs. 81.6%, *p* < 0.001), lower rate of de novo symptoms (1.2% vs. 16.5%, *p* =  < 0.001), higher postoperative resting pressure on manometry (*p* = 0.004), and higher postoperative squeeze pressure on manometry (*p* = 0.00) than PSR did. No significant difference was observed in obstructive defecation improvement (*p* = 0.238).

**Table 4:** Postoperative functional outcomes and patient satisfaction in the studied groups

	Group I (LVMR) (*n* = 250)	Group II (PSR) (*n* = 80)	*p* value
Recurrence of prolapse,* n* (%)	5 (2%)	16 (20%)	< 0.001*
Length of recurrence (mm), mean ± SD	69 ± 9	96 ± 14	< 0.001*
Surgery–recurrence interval (months), mean ± SD	40 ± 10	13 ± 9	< 0.001*
Baseline Wexner score, median (range)	15 (12–18)	15 (12–17)	0.010*
Wexner incontinence score, median (range)			
At 3 months	1 (0–6)	2 (0–6)	0.023*
At 4 years	1 (0–13)	2 (0–14)	0.030*
Baseline Altomare score, median (range)	22 (12–32)	22 (12–30)	0.270
Altomare score, median (range)			
At 3 months	0 (0–8)	1 (0–12)	0.025*
At 4 years	0 (0–22)	1 (0–24)	0.047*
Fecal incontinence improvement,* n* (%)			
Improved	106 (95.5%)	31 (83.8%)	
Initial improvement then worsening	5 (4.5%)	6 (16.2%)	0.019*
Obstructive defecation improvement,* n* (%)			
Improved	48 (92.3%)	14 (82.4%)	
Initial improvement then worsening	4 (7.7%)	3 (17.6%)	0.238
Development of new (de novo) symptoms,* n* (%)			
New constipation	3 (1.2%)	7 (9%)	
Urge to defecate	0	6 (7.5%)	< 0.001*
Postoperative manometry,* n* (%)	144 (57.6%)	52 (65%)	0.21
Resting pressure, mmHg	82 (46–101)	79 (34–87)	0.004*
Maximum squeeze pressure, mmHg	113 (100–200)	108 (85–130)	0.000*
Patient satisfaction,* n* (%)			
Satisfied	242 (98%)	62 (81.6%)	
Not satisfied	5 (2%)	14 (18.4%)	< 0.001*

Univariate logistic regression analysis was done for all variables, as shown in Table 5. ASA score, comorbidities, dolichocolon, and intraoperative complications were not eligible for regression analysis because of zero frequencies when classified according to the outcome, interfering with regression iterations and reaching the maximum number without a final solution. Only significant variables on the univariate level and variables that are known to be clinically relevant were included in a stepwise multivariate logistic regression model. The stepwise method was used as we have only 21 patients with recurrence; therefore, the model would not accommodate many predictors. After stepwise regression analysis, the only predictors that remained in the model were duration of symptoms (1-month increased duration was associated with 11% increased risk of recurrence; OR 1.11, 95% CI 1.012–1.217, *p* = 0.027), length of prolapse (1-cm increased length was associated with 40.7% increased risk of recurrence; OR 1.407, 95% CI 1.197–1.655, *p* < 0.001), and group I (LVMR), which was associated with 88.2% risk reduction of recurrence (OR 0.118, 95% CI 0.019–0.714, *p* = 0.02).

**Table 5:** Univariate and multivariate logistic regression analyses to predict rectal prolapse recurrence

	Univariate		Multivariate	
	OR (95% CI)	*p* value	OR (95% CI)	*p* value
Age (years)	0.963 (0.89–1.043)	0.355	–	–
Sex (ref: male gender)	1.132 (0.368–3.48)	0.828	–	–
Parity (ref: nulliparous)	1.274 (0.16–10.165)	0.819	–	–
BMI	0.994 (0.918–1.076)	0.883	–	–
Duration of symptoms (months)	1.097 (1.051–1.145)	< 0.001*	1.11 (1.012–1.217)	0.027*
Length of prolapse (cm)	1.502 (1.334–1.692)	< 0.001*	1.407 (1.197–1.655)	< 0.001*
Diabetes mellitus	3.006 (0.988–9.142)	0.053	–	–
Coronary diseases	4.479 (1.023–19.605)	0.047*	–	–
Initial symptoms			–	–
Prolapse with incontinence	4.135 (1.167–14.653)	0.028*	–	–
Prolapse with OD	1.667 (0.327–8.499)	0.539	–	–
Preoperative manometry	0.613 (0.251–1.498)	0.283	–	–
Operative time (min)	0.978 (0.962–0.993)	0.005*	–	–
Blood loss (ml)	1.012 (1.007–1.018)	< 0.001*	–	–
Group I	0.082 (0.029–0.231)	< 0.001*	0.118 (0.019–0.714)	0.02*
Baseline Wexner score	0.841 (0.547–1.294)	0.432	–	–
Baseline Altomare score	1.001 (0.825–1.215)	0.993	–	–

*OR* odds ratio, *95% CI* 95% confidence interval

*Significant *p* value

**Fig. 7:**
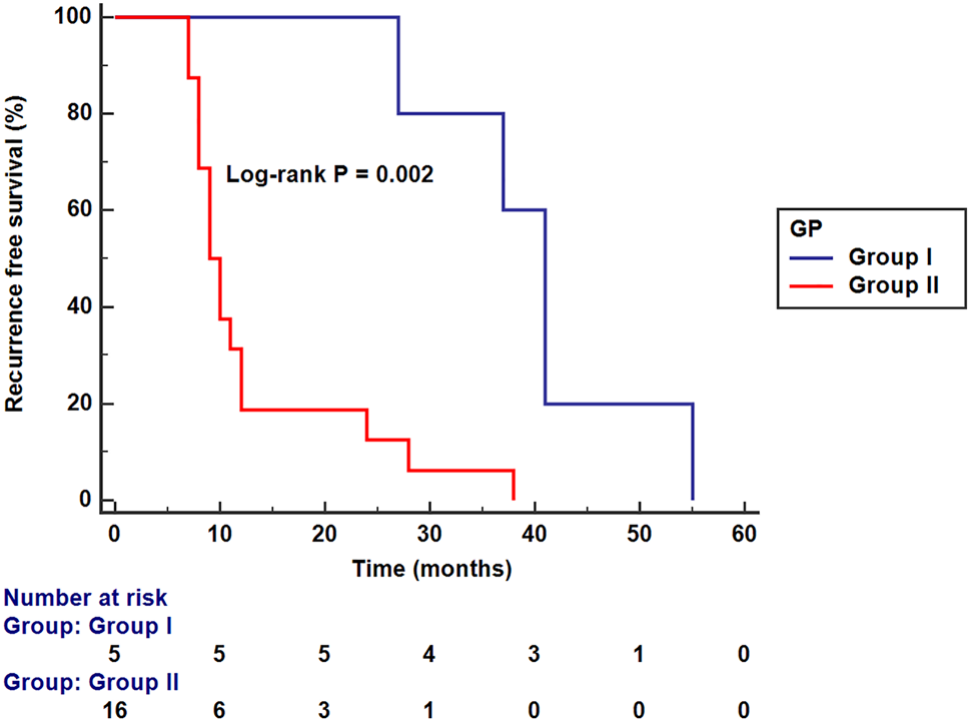
Kaplan–Meier analysis showing median recurrence-free survival time

Kaplan–Meier analysis revealed that the median recurrence-free survival time was significantly longer in the LVMR group (median, 41 months; 95% CI 37.494–44.506) than in the PSR group (median, 9 months; 95% CI 7.432–10.568) (log-rank *p* = 0.002) (Fig. 7).

## Discussion

To the best of our knowledge no previous study has compared the recurrent prolapse (RP), FI, and ODS after LVMR and PSR in elderly patients. This study demonstrated that LVMR was an efficacious intervention for managing EFTRP in elderly patients, with a favorable profile of low complication rates, reduced recurrence rate, and acceptable postoperative functional outcomes. LVMR demonstrates superior benefits compared to PSR in elderly patients with EFTRP.

The risk of RP following LVMR varies depending on the study design, characteristics of the studied population, and duration of follow-up time [26], with recurrent incidence of 2.8% [4], 9.6% [27], and 6.6% [28], respectively. The incidence of recurrent prolapse during follow-up after LVMR (5/250, 2%) in our study was lower than previously reported. At the same time, the RP after PSR in our study was reported to be 20% (16/80). A previous report showed that incidence of recurrent prolapse increased during long-term follow-up [29], similar to the data in our study. Several factors may have contributed to the lower incidence of recurrent prolapse after LVMR in our study. First, we strictly defined recurrence to include only EFTRP and no other forms of recurrence, such as mucosal prolapse or internal intussusception [30]. The second factor is the use of polypropylene mesh, which has a low incidence of recurrence. In fact we ensure a standardized width and length mesh, fixed to the ventral rectal wall with two rows of stitches, placed in the mid rectum, as low as possible to the pelvic muscles plane which minimized recurrence [31]. We used synthetic non-absorbable polypropylene mesh because of the cost of biological mesh from one side, and we covered the mesh with the peritoneum to avoid mesh exposure and subsequent complications. Glasgow et al. [32] found that recurrence following perineal surgery was related to prolapse of more than 1 year before surgery. In our study, recurrence is higher in PSR than LVMR, probably as a result of many factors: dolichocolon was higher in the PSR than in the LVMR group. The PSR does not require fixation of the long colon and has a higher recurrence rate [18]. The duration of symptoms before surgery and prolapse length were other factors that caused a higher recurrence of PSR. Another factor that increased the incidence of RP after PSR was an underlying weakness in the sphincter mechanism (evidenced by preoperative and postoperative Wexner scores) or because the presence of prolapse protruding for a longer time in PSR than in LVMR through the anal canal leads to a poor sphincter function [33]. Male sex, mesh length [4], old age, and poor preoperative continence [31] are risk factors for RP after LVMR. Our multivariate logistic regression analysis showed that recurrent prolapse was associated with PSR, similar to the results of a previous study [34]. Additionally, prolonged symptom duration before surgery and preoperative prolapse length were risk factors for recurrent prolapse. Furthermore, PSR is associated with earlier prolapse recurrence than LVMR.

ODS and FI are prominent symptoms associated with rectal prolapse [35]. Numerous studies have indicated that LVMR is more effective than alternative approaches for addressing FI [36]. EFTRP may result in anal sphincter stretching, compromising sphincter integrity and leading to FI [37]. Regarding ODS, our findings align with a previously published study [38], demonstrating that both LVMR and PSR approaches contribute to ODS improvement, although only LVMR repair reached statistical significance based on the Altomare score. Additionally, the two groups showed significant differences in patient satisfaction, reinforcing the value of the LVMR approach. LVMR has gained popularity owing to its autonomic nerve-sparing technique, which results in favorable functional outcomes and low morbidity and RR [39]. During follow-up, there was a significant improvement in preoperative FI and ODS in favor of LVMR compared with PSR, and the improvement was significant in FI in LVMR (106/111, 95% vs. 31/37, 83.8%, *p* = 0.01), while improvement in ODS did not reach a significant difference in either group (48/52, 92.3% vs. 14/17, 82.4%, *p* = 0.238). Wexner incontinence and Altomare scores were better 3 months postoperatively in the LVMR group and continued to improve significantly until the end of the study. Our data provide adequate evidence supporting the efficacy and reliability of LVMR for the treatment of EFTRP compared to PSR [30, 40]. Consten et al. reported that among patients who underwent LVMR, 63% (62 out of 98) experienced improvements in FI (FI), and 60% (50 out of 82) showed improvements in ODS. The median follow-up period was 34 months [39]. Similarly, other studies have also demonstrated favorable outcomes following LVMR, with reported improvements in FI ranging from 70% to 90% and ODS ranging from 60% to 80% [38, 41]. In our assessment for LVMR, although there were differences in symptom improvement compared to other studies, postoperative FI showed a significant improvement of 95.5% at the 4-year follow-up, whereas ODS demonstrated a significant improvement of 92.3%. These findings are consistent with another study [42]. The observed improvement in ODS symptoms after LVMR can be attributed to the autonomic nerve-sparing surgical technique [9]. Furthermore, this procedure facilitates the restoration of rectal anatomy, enhanced physiological function of the rectum, restored sensitivity to feces, and improved FI symptoms [43]. The improved outcomes observed in our centers can be attributed to the establishment of specialized multidisciplinary pelvic floor units staffed by skilled surgeons proficient in pelvic floor surgery; an increase in awareness among both patients and physicians regarding rectal prolapse and its treatment options was also important. Incidence rates of de novo OD and FI after LVMR have been reported to be 3.7% and 6.0%, respectively [38, 39]. Our present study showed new-onset symptoms in 3 patients (3/250, 1.2%) after LVMR and 13 patients (13/80, 16.5%) after PSR. Three patients (1.2%) developed new constipation following LVMR, while urge to defecate and constipation were observed in six (7.5%) and seven (9%) after PSR, respectively. PSR has been associated with an increased risk of urgency, which is attributed to a reduced rectal ampulla volume. A study reported a 1-year incidence of 26.8% among patients in the European Stapled Transanal Rectal Resection Registry [44]. By contrast, our LVMR study found no cases of postoperative urgency. This absence of urgency is likely due to the absence of rectal resection in the LVMR. The primary advantage of LVMR is the surgeon’s ability to minimize rectal mobilization and avoid lateral dissection, potentially leading to ascending parasympathetic sacral nerve damage. Such damage could result in denervation of left colon and rectum with inertia and the subsequent new onset of constipation [45]. In our study, new constipation developed after LVMR, possibly due to associated rectal stenosis that improved with dilation or due to an associated dolichocolon. In our study, we did not perform a resection of the redundant colon. However, resection of the redundant colon significantly reduces constipation [46]. The limitations of this study include its retrospective design and small sample size (which may have introduced selection bias). Postoperative sexual function changes were not collected. The relatively short-term follow-up is another limitation. Follow-up predominantly relied on telephone interviews, with fewer elderly patients evaluated in clinical settings. The absence of complete data on preoperative and postoperative manometry for all patients is a limitation of this study. We depended on clinical evaluation scores, namely the Wexner and Altomare scores, in postoperative evaluation rather than manometric or radiological evaluation. Additionally, this study evaluated one form of recurrent rectal prolapse which is EFTRP and did not include other forms of prolapse such as partial rectal prolapse and internal rectal prolapse. Although we recognize the existing body of evidence favoring LVMR, we believe that our study makes a valuable contribution by refining and contextualizing these findings in the current clinical landscape. The field of rectal prolapse surgery in the elderly is dynamic and surgical techniques are constantly evolving. Our study provides contemporary evidence considering recent advancements in surgical approaches and contributes to the current understanding of the comparative effectiveness of LVMR and PSR. Our study adds value by offering insights into the clinical implications and considerations when selecting between LVMR and PSR in certain situations. This information may be particularly relevant to clinicians who make decisions in real-world practice.

## Conclusions

LVMR has proven to be an efficacious intervention for managing EFTRP in elderly patients, with a favorable profile of low complication rates, reduced RR, and acceptable postoperative functional outcomes. LVMR demonstrates superior benefits compared with PSR in elderly patients with EFTRP.

## Data Availability

The datasets analysed during the current study are available from the corresponding author on reasonable request.
